# Transformation of heavy metals from contaminated water to soil, fodder and animals

**DOI:** 10.1038/s41598-024-62038-7

**Published:** 2024-05-22

**Authors:** Hina Kanwal, Ali Raza, Muhammad Saqlain Zaheer, Muhammad Nadeem, Hafiz Haider Ali, Salim Manoharadas, Muhammad Rizwan, Muhammad Saleem Kashif, Uzair Ahmad, Kamran Ikram, Muhammad Waheed Riaz, Fatima Rasool

**Affiliations:** 1 Department of Zoology, University of Sialkot, Punjab, Pakistan; 2 Department of Agronomy, University of Sargodha, Punjab, Pakistan; 3 Department of Agricultural Engineering, Khwaja Fareed University of Engineering and Information Technology, Rahim Yar Khan, Pakistan; 4 Institute of Food Science and Nutrition, University of Sargodha, Punjab, Pakistan; 5 Department of Agriculture, Government College University Lahore, Lahore, Punjab, Pakistan; 6 Department of Botany and Microbiology, College of Science, King Saud University, 11451 Riyadh, Saudi Arabia; 7 Department of Plant Nutrition, Institute of Crop Science and Resource Conservation (INRES), University of Bonn, 53115 Bonn, Germany; 8 Soil Testing Laboratory, Division of Agriculture, University of Arkansas, Fayetteville, USA; 9 State Key Laboratory of Wheat Breeding, Group of Wheat Quality and Molecular Breeding, College of Agronomy, Shandong Agricultural University, Tai’an, 271000 Shandong China; 10 Department of Bioinformatics, The Islamia University of Bahawalpur, Bahawalpur, 63100 Pakistan; 11 Department of Plant Sciences, Aberdeen Research & Extension Center, University of Idaho, Aberdeen, Idaho, USA

**Keywords:** Waste water irrigation, Pollution, Heavy metals, Animal fodder, Milk contamination, Plant sciences, Environmental sciences

## Abstract

A serious environmental problem that threatens soil quality, agricultural productivity, and food safety is heavy metal pollution in water sources. Heavy metal pollution is the main problem in tehsil Pasrur, Sialkot, Pakistan. Present study was arranged to notice the heavy metals in water, soil, forages and buffalo milk. There are seven sites that were used for this experiment. Highest malondialdehyde (MDA) contents (3.00 ± 0.01) were noticed in barseem roots at site 7. Ascorbate Peroxidase (APX) was reached at its peak (1.93 ± 0.01) at site 7 in the fresh barseem. Maximum protein contents (0.36 ± 0.01) were observed in fresh plant samples at site 2. Site 3's buffalo milk samples had the highest Ni content (7.22 ± 0.33 ppm), while Site 3's soil samples had the lowest Cr content (8.89 ± 0.56 ppm), Site 1's plant shoots had the lowest Cr content (27.75 ± 1.98 ppm), and Site 3's water had the highest Cr content (40.07 ± 0.49 ppm). The maximum fat content (5.38 ± 2.32%) was found in the milk of the animals at site 7. The highest density (31.88 ± 6.501%), protein content (3.64 ± 0.33%), lactose content (5.54 ± 0.320%), salt content (0.66 ± 0.1673%), and freezing point (− 0.5814 ± 0.1827 °C) were also observed in the milk from animals at site 7, whereas site 5 displayed the highest water content (0.66 ± 0.1673%) and peak pH value (11.64 ± 0.09). In selected samples, the pollution load index for Ni (which ranged from 0.01 to 1.03 mg/kg) was greater than 1. Site 7 has the highest conductivity value (5.48 ± 0.48). Values for the health risk index varied from 0.000151 to 1.00010 mg/kg, suggesting that eating tainted animal feed may pose health concerns. Significant health concerns arise from metal deposition in the food chain from soil to feed, with nickel having the highest health risk index.

## Introduction

Pakistan has a 34.4 square kilometer area dedicated to agriculture. Nath et al.^1^ and Nagajyoti et al.^2^ reported that the breeding land is regarded as agronomical land that enhances the production of plants and grazing land. In Sialkot, there are 3229 industrial gadgets; over 250 are dye-work equipment, producing 547–814 m^3^ of tannery waste water in step with day that leads to extreme environmental issues^3^. Many heavy metals such as mercury (Hg), cadmium (Cd), chromium (Cr), lead (Pb), copper (Cu), nickel (Ni), and zinc (Zn) are known as the toxic properties, causing the environmental risks and significantly damaging the human health^4,5^. Diverse natural processes such as volcanic eruptions, mineral-laden spring waters, metal structure corrosion, and microbial activity in soil and water can produce these metals^6,7^. Industrial processes such as mining, manufacturing and combustion are also playing the main role in spreading of these dangerous metals in the environment^8^. Due to modern industrialization, Lead (Pb) and cadmium (Cd) toxicity significantly damaging the plant, animals and human health and air pollution are also the main cause of this spreading toxic atmosphere. Long-term presence of heavy metals in the atmosphere, soil or water can cause the bio-accumulate, and potential for bio-magnification in food chains that directly damage the ecology and health on the earth^9^. Poor food quality is a problem but food with high heavy metal concentration can damage the whole life system and can cause severe diseases so its presence in the crops and food products should be minimize. Heavy metal pollution also negatively effects on biodiversity, ecosystem health and the viability of agricultural practices. Understand the toxic pathway and mechanism for enhancing mitigation strategies of heavy metals can be the safeguard for both environment integrity and human well-being^8,9^. The presence of several agricultural soil borne diseases are caused by airborne bacteria, toxic water, chemicals, and flora that enhance the absorption of heavy metals into the food chain, this is also threatening human health and animals^12^.Examiners in Pakistan are worried about the amount and concentration of exposure to these toxins because of the health risks they pose to most people^3^. Water is an essential resource for domestic, commercial, and agricultural use that accounts for 20% of the world's water supply; despite making up just 0.61% of the world’s total water reserves^10,11^. Despite providing a relatively germ-free supply of drinking water, various pollutants can enter the groundwater. Due to anthropogenic activities and herbal effects, there is a trade in either groundwater or surface water^3^. Farmers don't use wastewater as effectively as they could because the people who make and suggest network coverage solutions need to think differently and come up with creative ideas^6^. Metals are present on earth consisting of the life; from 35 metals present in herbs, 23 own high precise density greater than 5 g/cm^3^ with nucleon number rose above 40.04 called thrash metals. In cities, atmosphere contamination has been enhanced due to tanneries waste materials considered as municipal extracts^12^.

Chromium and cadmium amounts in textile wastewater are typically observed to be greater than the maximum likely to have at least in Pakistan^3^. Frequent monitoring of these heavy metals in tannery effluents is necessary, as are corrective actions, to defend both the ecosystem and life in tannery sites^12^. Maximum content of organic residue are located in the soil's top surface as in rural fields’ adjacent towns or commercial sites are monoculture crops, water is used for water storage. The upper soil layer does indeed have a serious influence on the materials that can be absorbed by flora from the soil although many crops' roots develop in this area^10^.

Sheep, goats, cows, and buffaloes digest the plant-based diet by progressively dissolving it in the rumen, which is the animal's initial stomach are profoundly different from other ruminants^13^. Dairy foods are essential parts of the human diet. Considering consumption increases essential elements in balanced concentrations along with proteins, significant Vitamins, minerals, lactose, and fatty acids, it has been described to as a complete diet^14^. Heavy metals in forages have really been implicated in a variety of sicknesses, physiological issues, and an imbalance in nutrient concentration^13^. Plants typically uptake all essential nutrients from soil and, the tonicity of a plant is greatly related to the frigidity of the soil^3^. Increased environmental exposure has made milk infection queries surrounding milk properties stronger. Residential or commercial effluents, combustion, bushfires, the dissolution of synthetic fertilizers, pesticides, etc. are the main sources of metal toxicity for humans^13^. This untreated water is used for growing crops in many states^14^. Because of this heightened knowledge of the heavy metals prevalent in flora, which affect human and animal health through the food chain^13,14^.

The main objectives of this study were (1) to observe the permissibility level and pollution severity of heavy metals in Tehsil Pasrur and Sialkot and (2) to investigate absorption of heavy metals in water, transfer to soil, forages and then to buffalo milk and was to examine the potential life threat due to intake of heavy metals. The importance of heavy metal contamination remediation techniques was emphasized by this research, which also highlights the connections between environmental health, agricultural sustainability and food security. This work offers a strong framework for tackling the problems associated with pollution by following the paths by which heavy metals move from water to soil, forages and animal products. These discoveries represent a substantial contribution to environmental science and provide stakeholders and policymakers with useful information that will help safeguard ecosystems and public health in impacted areas.

## Materials and methods

**Figure 1:**
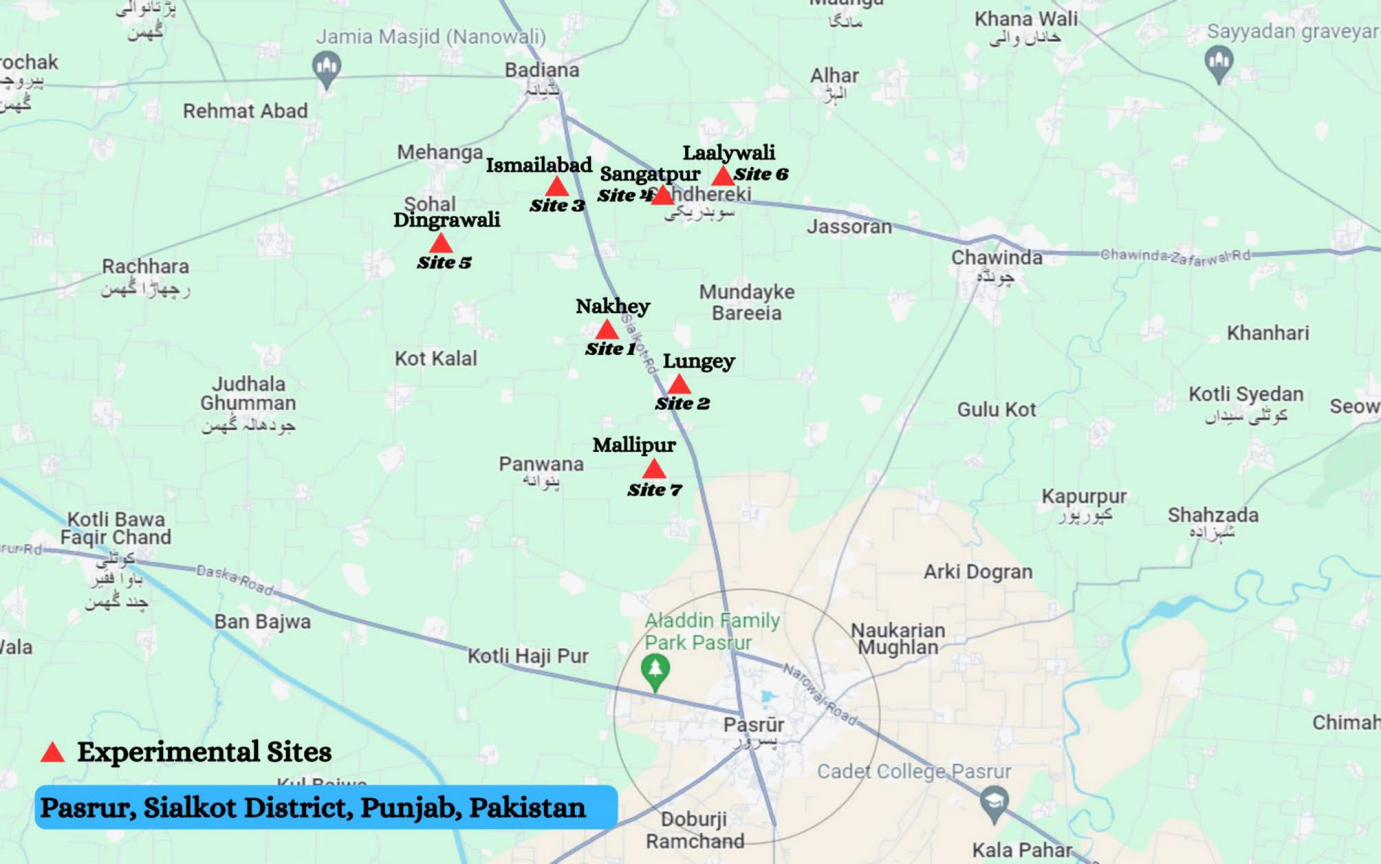
Experimental Sites for data collection. Pasrur, Sialkot District, Punjab, Pakistan (32°16′0N 74°40′0E).

Pasrur is very well known industrial city divided into 26 wards situated in Pakistan's Punjab province and yet is centered in the Sialkot District. Samples were gathered at the end of winter season 2022. Seven distinct sites from Pasrur, Sialkot (Nakhey (Site 1), Lungey (Site 2), Ismailabad (Site 3), Sangatpur (Site 4), Dingrawali (Site 5), Laalywali (Site 6), Mallipur (Site 7)) were chosen for soil, fodder and milk sampling because soil, water, fodder and milk of these areas is heavily accumulated by hazardous metals (Fig. 1). As per location 3 samples of soil and fodder (Barseem) while 5 samples of milk and total 77 samples from all sites were gathered.Soil samples were collected from fields where animal fodder had been harvested and 3 replicates of soil from 3 different parts of each field were randomly chosen for sampling from the very start of fields, the middle of the field, and the last edge of the field, respectively. Sampling was also discriminated on base of their depth for example; 1st sample was obtained from top surface, 2nd from 6 cm depth and 3rd was collected from 12 cm depth then packed into labeled plastic zipper bags. Same as fodder samples from seven sites with 3 replicates from each location were assembled and placed in proper packing in cool temperature till analysis. For milk sampling buffaloes from same sites were selected but number of milk sample replicates were increased as milk taken from 5 buffaloes each site and total 35 milk samples from seven sites were obtained from 35 buffaloes.

### Soil samples analysis

The dried soil samples were subjected to moisture removal by placing them in an oven at 79 °C for 72 h. The samples underwent digestion using a mixture of concentrated sulfuric acid (H_2_SO_4_). Initially, 5 ml of concentrated H_2_SO_4_ was added to the soil samples, followed by the addition of 8 ml of H_2_SO_4_. The mixture was placed in a digestion chamber for 30 min at room temperature. After this initial digestion step, an additional 3 ml of H_2_SO_4_ was added to the mixture and the samples were heated in a digestion chamber at temperatures ranging between 95 °C to 130 °C. The heating process continued until brown fumes were observed, indicating the completion of digestion and the formation of colorless solutions. Following digestion, the samples were cooled for 4–5 min and then the mixture was diluted by adding 50 ml of distilled water. The diluted mixture was filtered using Whatman filter paper No. 42 mm to remove any insoluble particles and the filtrate was collected in labeled plastic clean bottles for further analysis.

### Fodder samples analysis

Barseem plant samples (fodder) were initially washed with water 1st and again washed in laboratory with dilute hydrogen chloride (HCl) and placed the samples in shad to dry. When samples were dried then put them in incubator for 72 h (three days) to make them drier and moisture free. Digestion of plant samples was done with 2 g of root and 2 g of shoot in 4 ml of H_2_SO_4_ and 4 ml of hydrogen peroxide separately. Samples were placed in hot plates at 130 °C for 30 min then cool at normal temperature. Added 2 ml of hydrogen peroxide in each sample and did this same procedure until samples became colorless after brown fumes. Added 50 ml of distilled water in each sample and filtered them in clean labeled plastic bottles.

### Milk samples analysis

Collected 35 buffalo milk samples from seven locations of Tehsil Pasrur, District Sialkot, as gathered five bottles of milk from five different buffalo animals from each location in tagged, washed, and cleaned plastic bottles. Using a pipette, extract 0.5 mL of milk from each milk sample and poured it into plastic bottles with perchloric acid and hydrochloric acid solutions, respectively, and frozen them until the next step. After that poured those samples into 200 ml beakers and covered all the beakers with aluminum sheets properly, placed on hot plates at 130 °C. When samples were put on hot plates and 2–3 min were passed, samples started to release brown fumes initially then those brown fumes turned to white fumes after 4–5 min. A pungent odor was detected, along with white fumes, and samples were removed from hot plates using tongs or cotton pieces and placed at room temperature(add the temperature) until they cooled. Filtered all of the samples and then pipetted 50 ml of distilled water into each one to prepare the samples for heavy metal detection in spectrophotometry (720D China) and store them in the laboratory with proper labeling.

### Metal analysis and standards preparation

Atomic absorption spectrometer was used to determine the heavy metals concentration in soil, water and fodder as reported by Lemessa et al.^15^ and flame atomic absorption spectrophotometry (FA-AAS) method was used to determine the heavy metals in the animal milk as reported by Sikirić et al.^16^. In distinguishing examination work, values of Ni, Cd, Cu and Pb were analyzed by using their specific standard curves (BDH atomic absorption standards). The glassware was carefully cleaned in order to create a standard solution. In order to get rid of any metal ions those might have absorbed from anywhere. We immersed them in 30% nitric acid for two hours and then gave them a good rinse under running tap water and allowed them to air dry in a dry environment. We thoroughly cleaned and rinsed the funnel and flask with distilled water.

### Milk related parameters

The Lactoscan milk analyzer uses the most recent, top-notch ultrasonic technology. The basis for the ultrasonic working principle is the measurement of the ultrasound's velocity in milk and dairy products. Since all Lactoscan milk analyzer results are based on precise measurements of the parameters, they may be trusted. Some of the fundamental drawbacks of infrared technology are overcome by ultrasound technology. Using the Lactoscan Ultrasonic Milk Analyzer, ultrasound was used to determine milk composition. Milk samples before use (2 ml) were warmed to room temperature (31˚C). Milk analysis indicated salt content, pH, freezing point, fat, protein, lactose, and salt percentages, as well as the presence of additional water were also assessed with ultrasonography. Occasionally, when using ultrasonic analysis, water irregularities such as high percentages and erratic freezing point may be noticed.

Toxic heavy metals enter the body of organisms through different pathways. It may be through skin contact, during breathing, and/or by consuming contaminated fodder etc. Daily intake of metal was calculated by following formula given by Sarwar et al.^17^.

$$DIM=\frac{(\mathrm{C \,meta}\times \mathrm{D \,food \,intake})}{(B \,average \,weight)}$$

where: DIM expressed the daily intake of metal.

C is the heavy metal concentration in the fodder (mg/kg).

D is the daily intake of the fodder by sheep (mg/kg).

B is the average weight of sheep (kg).

An indicator of the possible health risks associated with environmental pollution or toxins is the Health Risk Index (HRI). To assess whether there may be a risk to human health, it compares the actual dose intake of a substance (DIM) to a reference guideline value (RFG). The values of HRI depend on the daily intake of metals and oral reference dose. Health risk index was determined by using formula described and procedure reported by Khan et al.^18^ and Cao et al.^19^.

$$HRI=\frac{{\text{DIM}}}{RFG}$$

Enrichment factor had been determined by Cui et al.^20^.

$$EF=\frac{([{\text{M}}]{\text{FodderE}}/[{\text{M}}]{\text{SoilsE}})}{([M]FodderS/[M]SoilsE)}$$

where:

EF indicates enrichment factor.

[M]FodderE indicates the heavy metals concentration in examined fodder sample.

[M]SoilE indicates the heavy metals concentration in examined soil sample.

[M]FodderS indicates the standard values of heavy metals in fodder.

[M]SoilS indicates the standard values of heavy metals in soil.

Bio concentration factor is the transfer of metal from soil to plant, it was measured by using Microsoft Excel, by using following formula reported by Cui et al.^20^.

$$BCF=\frac{\left[{\text{M}}\right]\mathrm{Fodder \,samples}}{(\left[M\right]FodderSamples)}$$

where: BCF expressed for bio concentration factor for fodder/ soil samples.

[M] Fodder is the total metal concentration (mg/kg) in fodder samples.

[M] Soil is the total metal concentration (mg/kg) in the soil from where samples of fodder were collected.

PLI parameter was measured by following formula as given by Cui et al.^20^.

$$PLI=\frac{\left[{\text{M}}\right]{\text{IS}}}{(\left[M\right]RS)}$$

where: ‘PLI stands for pollution load index for soil’.

[M]IS ‘shows heavy metal content in examined soil sample’.

[M]RS ‘shows metal concentration in reference soil’.

All experimental research and field studies involving plants, including the collection of plant material, were conducted in strict accordance with relevant institutional, national, and international guidelines and legislation. Prior approval was obtained from the appropriate authorities and all necessary permits were acquired.

### Statistical analysis

The correlation between each metal and soil, feed, water, and milk was calculated according to Liu et al.^21^ assessment, the significance of mean was at the 0.05 probability levels. The means of heavy metal samples were subjected to one-way analysis of variance (ANOVA). Association between soil, pasture, and milk in regards to the eviction of Ni and Cr were at 0.05 percentiles of probability, the importance of means was examined.

### Permission statement

Permissions were obtained to collect milk sample from the relevant authorities.

## Results

### Metal analysis

The soil samples we gathered from seven different sites showed notable differences in nickel (Ni) content, with a significance level of *p* < 0.01. We found the highest amount of Ni in the soil samples from site 3, measuring at 17.57 ± 0.89 ppm. Conversely, the lowest Ni concentration was observed in samples taken from site 5, with a measurement of 15.06 ± 0.73 ppm, as detailed in Table 1.We gathered fresh Barseem plants along with their roots from specific locations to study how much nickel they contained. We noticed a significant difference (*p* < 0.01) in nickel levels among these sites. The highest nickel content was found in roots from site 4, measuring 11.22 ± 0.27 ppm. On the other hand, the lowest nickel content was observed in roots from site 6, with a measurement of 4.83 ± 0.88 ppm, as detailed in Table 1. The plant shoots from site 6 had the highest Ni content, measuring 81.18 ± 1.77 ppm, which was the maximum value among all samples. In contrast, the lowest Ni content was found in shoot samples from site 4, with a measurement of 6.53 ± 0.38 ppm. The milk samples from site 3 had the highest Ni content, measuring 11.04 ± 0.34 ppm. Conversely, the milk samples from site 1 had the lowest Ni content, with a measurement of 3.87 ± 0.36 ppm.

**Table 1:** Comparisons for Ni and Cr in irrigation soil, root, shoot of plants and milk of animals.

Site	Ni (Soil)	Ni (Root)	Ni (shoot)	Ni (Milk)	Cr (Soil)	Cr (Root)	Cr (Shoot)	Cr (Milk)	Pb (Shoot)
S1	16.23 ± 0.13abc	9.15 ± 0.42bc	9.21 ± 0.20b	3.87 ± 0.36c	3.25 ± 0.65de	7.07 ± 1.27c	27.75 ± 1.98a	9.03 ± 0.15c	ND
S2	17.18 ± 0.27ab	10.32 ± 0.66ab	8.39 ± 0.38b	10.46 ± 1.29a	7.25 ± 0.41b	6.52 ± 1.16c	9.16 ± 0.64cd	26.94 ± 1.48a	0.517 ± 0.208b
S3	17.57 ± 0.89a	10.98 ± 0.21a	7.71 ± 0.96b	11.04 ± 0.34a	2.78 ± 0.87de	1.62 ± 0.34e	21.94 ± 1.13b	5.70 ± 0.66d	0.863 ± 0.118b
S4	15.16 ± 0.17c	11.22 ± 0.27a	6.53 ± 0.38b	7.97 ± 0.23b	8.89 ± 0.56a	47.40 ± 2.35a	9.90 ± 0.43cd	3.30 ± 0.85d	0.479 ± 0.114b
S5	15.06 ± 0.73c	8.93 ± 0.06bc	7.02 ± 1.33b	6.63 ± 0.45b	1.80 ± 0.34e	2.10 ± 0.54de	8.04 ± 1.92d	13.65 ± 1.14b	0.790 ± 0.120b
S6	16.76 ± 0.50ab	4.83 ± 0.88d	81.18 ± 1.77a	9.78 ± 0.48a	3.92 ± 0.35cd	15.50 ± 0.84b	9.87 ± 1.77cd	0.20 ± 0.09e	5.730 ± 0.960a
S7	15.92 ± 0.32bc	8.03 ± 0.70c	9.11 ± 0.73b	6.97 ± 0.14b	4.97 ± 0.12c	5.16 ± 1.06cd	12.94 ± 1.44c	8.82 ± 1.60c	0.063 ± 0.071b

Means that do not share a letter are significantly different.

The soil samples from site 4 had the highest concentration of Cr, measuring 8.89 ± 0.56 ppm. Conversely, the soil samples from site 5 had the lowest Cr content, with a measurement of 1.80 ± 0.34 ppm. There was a notable difference in the amount of heavy metal accumulation between the values at site 4 and site 5, as detailed in Table 1. Plant root samples from seven specific sites to analyze the metal content, focusing on chromium (Cr). After analysis, we found a significant difference (*p* < 0.01) in Cr levels among these samples. The highest concentration of Cr was detected in root samples from site 4, measuring 47.40 ± 2.35 ppm. In contrast, the lowest Cr content was observed in soil samples from site 3, with a measurement of 1.62 ± 0.34 ppm. This indicates a notable variation in Cr levels across the different sampling locations. The peak value of Cr content was calculated from site 1 (27.75 ± 1.98 ppm) plant shoot samples. Milk samples from buffalo across seven different sites and discovered a significant difference (*p* < 0.01) in the chromium (Cr) content among them. The highest concentration of Cr was found in milk samples from site 2, with 26.94 ± 1.48 ppm. Conversely, the lowest Cr content was observed in milk samples from site 6, with 0.20 ± 0.09 ppm. The highest concentration of Pb was found in shoot samples from site 6, measuring 5.730 ± 0.960 ppm. Interestingly, no detectable Pb content was found in the shoot samples collected from site 1.

### Milk analysis

Milk samples collected from seven different sites showed significant differences (*p* < 0.05) in various properties. For instance, fat content varied notably, with the highest concentration (5.38 ± 2.32%) found at site 7 and site 4 (4.96 ± 2.01%), while the lowest (2.70 ± 0.84%) was observed at Site 6, as detailed in Table 2. Similar variations were seen in Solid-Not-Fat (SNF) content, with the highest (9.18 ± 2.10%) at site 7 and the lowest (5.76 ± 0.39%) at site 5. pH levels also differed significantly (*p* < 0.05), peaking at site 5 (11.64 ± 0.09) and hitting a low at site 7 (11.40 ± 0.25). Protein content showed a range from the highest (3.64 ± 0.33%) at site 7 to the lowest (2.28 ± 0.31%) at site 5. Additionally, lactose, salt, water, freezing point, density, and conductivity content in the milk samples from the seven sites displayed notable differences (*p* < 0.05). For instance, the maximum lactose content (5.54 ± 0.320%) was seen in buffalo milk samples, while salt content peaked at site 7 (0.66 ± 0.1673%) and the lowest at sites 4 (0.46 ± 0.1140%) and 5 (0.46 ± 0.0894%). Water content ranged from a high of 25.66 ± 1.64% at site 5 to a low of 4.54 ± 6.12% at site 7. The freezing point, density, and conductivity values also varied significantly (*p* < 0.05) across sites, with site 7 consistently showing higher values compared to site 5, as outlined in Table 2.

**Table 2:** Comparison of mean values (± SD) of milk samples collected from different sites.

Site	Fat (%)	SNF (%)	Protein (%)	Lactose (%)	pH	Salt (%)	Conductivity	Added Water (%)	Density	Freezing Point (°C)
S1	3.02 ± 0.79	7.46 ± 0.23ab	2.48 ± 0.26a	4.28 ± 0.32b	11.54 ± 0.26	0.56 ± 0.0548	4.78 ± 0.13	5.22 ± 0.57d	26.34 ± 0.808ab	− 0.470 ± 0.0084ab
S2	3.80 ± 0.94	6.66 ± 1.19b	2.50 ± 0.42b	3.76 ± 0.60bc	11.52 ± 0.18	0.50 ± 0.1000	4.70 ± 0.01	20.40 ± 11.54a-c	22.30 ± 3.828b	− 0.419 ± 0.0765ab
S3	3.20 ± 0.75	6.56 ± 0.41b	2.44 ± 0.23b	3.50 ± 0.29bc	11.58 ± 0.13	0.50 ± 0.0707	4.52 ± 0.23	11.06 ± 6.12b-d	22.12 ± 1.834b	− 0.408 ± 0.0269a
S4	4.96 ± 2.01	7.20 ± 0.31b	2.58 ± 0.19b	3.90 ± 0.29bc	11.58 ± 0.21	0.46 ± 0.1140	4.42 ± 1.70	8.30 ± 5.32 cd	24.50 ± 0.686b	− 0.469 ± 0.0514ab
S5	3.16 ± 1.11	5.76 ± 0.39b	2.28 ± 0.31b	3.26 ± 0.27c	11.64 ± 0.09	0.46 ± 0.0894	4.56 ± 0.28	25.66 ± 1.64a	20.46 ± 1.553b	− 0.380 ± 0.0308a
S6	2.70 ± 0.84	6.16 ± 0.59b	2.36 ± 0.29b	3.16 ± 0.51c	11.54 ± 0.18	0.54 ± 0.1140	4.46 ± 0.25	23.52 ± 9.26ab	21.72 ± 2.649b	− 0.4026 ± 0.0619a
S7	5.38 ± 2.32	9.18 ± 2.10a	3.64 ± 0.33a	5.54 ± 0.320a	11.40 ± 0.25	0.66 ± 0.1673	5.48 ± 0.48	4.54 ± 6.12d	31.88 ± 6.501a	− 0.5814 ± 0.1827b

Means that do not share a letter are significantly different.

### Pollution indices (PLI, BCF, EF, DIM, HRI and Correlation)

Collected samples of water, soil, fodder, and animals from seven different sites in Tehsil Pasrur, Sialkot, and studied various parameters like BCF, DIM, PLI, HRI, EF, and correlation to analyze heavy metal accumulation. Each sample was analyzed statistically. The results for PLI in soil showed the highest value at Site 3 (1.9392) and the lowest at Site 4 (1.6732). For PLI Root, the highest value was at Site 4 (0.1674) and the lowest at Site 6 (0.0720), with others falling in between. The peak PLI (Shoot) value was at Site 6 (1.2116), while the lowest was at Site 5 (0.1047). Regarding BCF Root values, the highest was at Site 4 (0.7401) and the lowest at Site 6 (0.2881). For BCF (Shoot), the highest value was at Site 6 (4.8436) and the lowest at Site 5 (0.4661). EF values were tested in root and shoot samples, with the highest EF (Root) at Site 3 (1.1000) and the lowest at Site 6 (0.0389). EF (Shoot) peaked at Site 6 (19.100) and was lowest at Site 4 (1.6986). DIM (Root) had its peak at Site 4 (0.00506) and lowest at Site 6 (0.00217), while DIM (Shoot) peaked at Site 6 (0.002185) and lowest at Site 4 (0.000194). HRI (Root) showed the highest value at Site 4 (0.000151), and the lowest was at Site 5 (0.00012). HRI (Shoot) had its highest at Site 6 (0.07284) and lowest at Site 4 (0.00647) (Table 3).

**Table 3:** Comparisons for Ni and Cr in irrigation water, soil, root, shoot of plants and milk of animals.

Site	Ni (Water)	Ni (Soil)	Ni (Root)	Ni (shoot)	Ni (Milk)	Cr (water)	Cr (Soil)	Cr (Root)	Cr (Shoot)	Cr (Milk)	Pb (Shoot)
S1	7.22 ± 0.33a	16.23 ± 0.13abc	9.15 ± 0.42bc	9.21 ± 0.20b	3.87 ± 0.36c	27.80 ± 3.26b	3.25 ± 0.65de	7.07 ± 1.27c	27.75 ± 1.98a	9.03 ± 0.15c	ND
S2	5.78 ± 0.41ab	17.18 ± 0.27ab	10.32 ± 0.66ab	8.39 ± 0.38b	10.46 ± 1.29a	5.56 ± 0.27c	7.25 ± 0.41b	6.52 ± 1.16c	9.16 ± 0.64 cd	26.94 ± 1.48a	0.517 ± 0.208b
S3	5.70 ± 0.88b	17.57 ± 0.89a	10.98 ± 0.21a	7.71 ± 0.96	11.04 ± 0.34a	29.54 ± 5.32b	2.78 ± 0.87de	1.62 ± 0.34e	21.94 ± 1.13b	5.70 ± 0.66d	0.863 ± 0.118b
S4	5.09 ± 0.28b	15.16 ± 0.17c	11.22 ± 0.27a	6.53 ± 0.38b	7.97 ± 0.23b	8.17 ± 1.64c	8.89 ± 0.56a	47.40 ± 2.35a	9.90 ± 0.43 cd	3.30 ± 0.85d	0.479 ± 0.114b
S5	5.83 ± 0.34ab	15.06 ± 0.73c	8.93 ± 0.06bc	7.02 ± 1.33b	6.63 ± 0.45b	40.07 ± 0.49a	1.80 ± 0.34e	2.10 ± 0.54de	8.04 ± 1.92d	13.65 ± 1.14b	0.790 ± 0.120b
S6	6.29 ± 0.74ab	16.76 ± 0.50ab	4.83 ± 0.88d	81.18 ± 1.77a	9.78 ± 0.48a	27.92 ± 2.19b	3.92 ± 0.35 cd	15.50 ± 0.84b	9.87 ± 1.77 cd	0.20 ± 0.09e	5.730 ± 0.960a
S7	5.67 ± 0.25b	15.92 ± 0.32bc	8.03 ± 0.70c	9.11 ± 0.73b	6.97 ± 0.14b	8.78 ± 0.57c	4.97 ± 0.12c	5.16 ± 1.06cd	12.94 ± 1.44c	8.82 ± 1.60c	0.063 ± 0.071b

Means that do not share a letter are significantly different.

PLI (Soil) most elevated value presented at site 4 (0.9802) whereas the least possible value was tested at site 5 (0.1985). PLI (Root) highest value was examined at site 4 (20.609) and the merest value seen at site 3 (0.704) and the other value displayed in (Table 4). PLI (Shoot) values of collected samples of seven sites, the greatest value observed at site 1 (12.065) whereas the minimal value given at site 5 (3.496). BCF (Root) values were shown in (Table 4) and predicted the highest value at site 4 (5.332) and most bottom was measured at site 3 (0.583). BCF (Shoot) displayed the most prominent value at site 1 (8.538) while the lowest seen at site 5 (1.114). EF was measured in both root and shoot, maximum EF (Root) measured at site 4 (21.026) while the merest value observed at site 3 (2.298). EF (Shoot) the peak value discovered at site 1 (33.67) and most lowest value distinguished at site 4 (4.39). DIM in both root shoot shown values in (Table 4), according to the table the greatest value of DIM (Root) at site 4 (0.0213) and the minimum observed value at site 5 (0.0094) and site 3 (0.0073) with somehow differences in readings. DIM (Shoot) maximum value shown at site 1 (0.01252) and site 5 (0.00362) predicted the most bottom value shown in (Table 4). HRI (Root) given the most elevated value at site 4 (0.07128) while site 3 (0.0024) displayed the merest value and the other values were given in (Table 8) which were in between highest and lowest readings. HRI (Shoot) presented the highest value at site 1 (4.173) and the lowest value seen at site 5 (1.209) while the rest values were explained in (Table 4).

**Table 4:** Mean sum of square of pollution indices of Ni in fodder crops collected from different sites.

SOV	DF	PLI (Soil)	PLI (Root)	PLI (Shoot)	BCF (Root)	BCF (Shoot)	EF (Root)	EF (Shoot)	DIM (Root)	DIM (Shoot)	HRI (Root)	HRI (Shoot)
Sites	06	0.0339**	0.0032**	0.5120**	0.0581**	8.118**	0.001**	126.2**	0.000003^NS^	0.000002*	0.0000**	0.0018**
Error	14	0.00002	0.00002	0.00002	0.00002	0.00002	0.000002	0.000	0.000002	0.00000	0.0000	0.0000
Total	20											

** = Highly significant (*p* ≤ 0.01), * = Significant (*p* ≤ 0.05), ^NS^ = Non-significant (*p* ≥ 0.05).

Different metrics, including PLI, BCF, EF, DIM, and HRI, were used to correlate the soil and plant (root and shoot) data that had been obtained. PLI soil (− 0.025) had demonstrated a negative association with PLI root and a positive correlation with PLI shoot below (0.239). PLI root and PLI shoot revealed a negative correlation, measuring − 0.862. PLI soil and BCF root showed a negative association with PLI soil (− 0.288) and a positive correlation (0.964) with PLI root. Among other characteristics, PLI shot also showed a negative connection with BCF root (− 0.873). BCF shot's correlation with PLI soil and PLI shoot was positive (0.224, 1.000), whereas BCF shoot's correlation with PLI was negative (0.224, 1.000) similarly other parameters correlations values explained and given (Table 5).

**Table 5:** Mean sum of square of pollution indices of Cr in fodder crops collected from different sites.

SOV	DF	PLI (Soil)	PLI (Root)	PLI (Shoot)	BCF (Root)	BCF (Shoot)	EF (Root)	EF (Shoot)	DIM (Root)	DIM (Shoot)	HRI (Root)	HRI (Shoot)
Sites	06	0.2366**	148.58**	32.67**	9.800**	27.95**	152.41**	434.71**	0.0016**	0.0035**	0.0177**	3.908**
Error	14	0.00025	0.000	0.0000	0.0002	0.0000	0.000	0.000	0.0002	0.0000	0.0000	0.0000
Total	20											

** = Highly Significant (*p* ≤ 0.01), * = Significant (*p* ≤ 0.05), ^NS^ = Non-significant (*p* ≥ 0.05).

The results of applying correlation to various parameters were both positive and negative, with PLI shoot showing negative values (− 0.383 and − 0.295) in correlation with PLI soil and PLI root, respectively, while PLI root had a positive correlation value (0.750) with PLI soil. BCF showed positive correlations (0.516 and 0.907) with the PLI soil and root and negative correlations (− 0.237) with the PLI shoot. BCF shoot displayed both negative and positive values, with negative values (− 0.722, − 0.507, and − 0.403) in correlation with PLI soil, PLI root, and BCF root, while only PLI (0.886) presented a positive value same as other values of correlation among all described parameters given and explained with detail in (Table 6).

**Table 6:** Mean values of pollution indices of Ni in fodder crops collected from different sites.

Sites	PLI (Soil)	PLI (Root)	PLI (Shoot)	BCF (Root)	BCF (Shoot)	EF (Root)	EF (Shoot)	DIM (Root)	DIM (Shoot)	HRI (Root)	HRI (Shoot)
S1	1.7913d	0.1365b	0.1374b	0.5637d	0.5674b	0.0762d	2.2378c	0.004128	0.000256b	0.00012ab	0.00853c
S2	1.8962b	0.1540a	0.1252bc	0.6006c	0.4883c	0.0812bc	1.9258d	0.004655	0.000220b	0.000139a	0.00734d
S3	1.9392a	0.1638a	0.1150cd	0.6249b	0.4388e	0.0845b	1.7305f	0.004953	0.000198b	0.000148a	0.00659f
S4	1.6732f	0.1674a	0.0974e	0.7401a	0.4307e	0.1000a	1.6986g	0.005061	0.000194b	0.000151a	0.00647g
S5	1.6622e	0.1332bc	0.1047de	0.5929c	0.4661d	0.0801cd	1.8382e	0.004028	0.000210b	0.00012ab	0.00701e
S6	1.8498c	0.0720d	1.2116a	0.2881f	4.8436a	0.0389f	19.100a	0.002179	0.002185a	0.000065b	0.07284a
S7	1.7571e	0.1198c	0.1359b	0.5043e	0.5722b	0.0682e	2.2566b	0.003622	0.000258b	0.00010ab	0.00860b

Different small alphabetical letters show significant differences between treatments.

Correlation was applied on different parameters on collected data of soil and plant (root and shoot) such a PLI, BCF, EF, DIM and HRI. According to the (Table 8) given below, PLI soil (− 0.025) had shown negative correlation with PLI root and positive correlation with PLI shoot as (0.239). PLI root had given negative value (− 0.862) correlation with PLI shoot. PLI soil had presented negative value (− 0.288) correlation with BCF root and PLI root had mentioned positive value (0.964) correlation with BCF root. PLI shoot had also displayed negative value (− 0.873) correlation with BCF root among parameters. BCF shoot had shown positive correlation with PLI soil and PLI shoot as (0.224, 1.000) while negative values of BCF shoot were displayed negative in correlation with PLI root and BCF root, values are(− 0.865, − 0.873). EF root had shown negative correlation with PLI soil, PLI shoot and BCF shoot whose values are (− 0.288, − 0.873, − 0.873) while showed positive values in correlation with PLI root and BCF root as (0.964, 1.000). EF shoot showed negative values (− 0.865, − 0.873, − 0.873) in correlation with parameters PLI root, BCF root and EF root, respectively whereas positive values (0.224, 1.000 and 1.000) showed correlation with PLI soil, PLI shoot and BCF shoot. DIM root presented negative correlation as (− 0.025, − 0.865, − 0.865and − 0.865) with PLI soil, PLI shoot, BCF shoot and EF shoot while positive values (1.000, 0.964,0.964) of correlation with parameters PLI root, BCF root and EF root. DIM shoot showed negative values (− 0.865, − 0.873, − 0.873 and − 0.865) correlation with PLI root, BCF root, EF root and DIM root on the other hand, DIM shoot given positive readings (0.224, 1.000, 1.000 and 1.000) correlation with PLI soil, PLI shoot, BCF shoot and EF shoot, respectively (Table 8). When talking about HRI root, it had shown negative correlation values (− 0.025, − 0.862, − 0.865, − 0.865 and − 0.865) of parameters PLI soil, PLI shoot, BCF root, EF shoot and DIM shoot whereas parameters PLI root, BCF root, EF root and DIM root presented positive values (1.000, 0.964, 0.964 and 1.000). HRI shoot showed positive values (0.224, 1.000, 1.000, 1.000, 1.000) in correlation with PLI soil, PLI shoot, BCF shoot, EF shoot and DIM shoot whereas negative correlation values of (− 0.865, − 0.873, − 0.0873, − 0.865 and 0.865) of parameters PLI shoot, BCF root, EF root, DIM root and HRI root shown in (Tables 7, 8) given below.

**Table 7:** Mean values of pollution indices of Cr in fodder crops collected from different sites. **=Highly Significant (p≤0.01)*= Significant (p≤0.05)NS= Non-significant (p≥0.05).

Sites	PLI (Soil)	PLI (Root)	PLI (Shoot)	BCF (Root)	BCF (Shoot)	EF (Root)	EF (Shoot)	DIM (Root)	DIM (Shoot)	HRI (Root)	HRI (Shoot)
S1	1.7913d	0.1365b	0.1374b	0.5637d	0.5674b	0.0762d	2.2378c	0.004128	0.000256b	0.00012ab	0.00853c
S2	1.8962b	0.1540a	0.1252bc	0.6006c	0.4883c	0.0812bc	1.9258d	0.004655	0.000220b	0.000139a	0.00734d
S3	1.9392a	0.1638a	0.1150cd	0.6249b	0.4388e	0.0845b	1.7305f	0.004953	0.000198b	0.000148a	0.00659f
S4	1.6732f	0.1674a	0.0974e	0.7401a	0.4307e	0.1000a	1.6986g	0.005061	0.000194b	0.000151a	0.00647g
S5	1.6622e	0.1332bc	0.1047de	0.5929c	0.4661d	0.0801cd	1.8382e	0.004028	0.000210b	0.00012ab	0.00701e
S6	1.8498c	0.0720d	1.2116a	0.2881f	4.8436a	0.0389f	19.100a	0.002179	0.002185a	0.000065b	0.07284a
S7	1.7571e	0.1198c	0.1359b	0.5043e	0.5722b	0.0682e	2.2566b	0.003622	0.000258b	0.00010ab	0.00860b

Different small alphabetical letters show significant differences between treatments.

**Table 8:** Correlation Ni: PLI (soil), PLI (root), PLI (shoot), BCF (root), BCF (shoot), EF (root), EF (shoot), DIM (root), DIM (shoot), HRI (root), HRI (shoot).

	PLI (Soil)	PLI (Root)	PLI (Shoot)	BCF (Root)	BCF (Shoot)	EF (Root)	EF (shoot)	DIM (Root)	DIM (shoot)	HRI (Root)
PLI (Root)	− 0.025									
PLI (Shoot)	0.239	− 0.862								
BCF (Root)	− 0.288	0.964	− 0.873							
BCF (Shoot)	0.224	− 0.865	1.000	− 0.873						
EF (Root)	− 0.288	0.964	− 0.873	1.000	− 0.873					
EF (shoot)	0.224	− 0.865	1.000	− 0.873	1.000	− 0.873				
DIM (Root)	− 0.025	1.000	− 0.862	0.964	− 0.865	0.964	− 0.865			
DIM (shoot)	0.224	− 0.865	1.000	− 0.873	1.000	− 0.873	1.000	− 0.865		
HRI (Root)	− 0.025	1.000	− 0.862	0.964	− 0.865	0.964	− 0.865	1.000	− 0.865	
HRI (shoot)	0.224	− 0.865	1.000	− 0.873	1.000	− 0.873	1.000	− 0.865	1.000	− 0.865

After applying correlation on different parameters, the values obtained were both positive and negative as PLI root had shown positive value (0.750) of correlation with PLI soil and PLI shoot showed negative values (− 0.383 and − 0.295) in correlation with parameters PLI soil and PLI root. BCF given positive values (0.516 and 0.907) in correlation with PLI soil and PLI root while negative value (− 0.237) correlation with PLI shoot. BCF shoot presented both negative and positive values as negative values (− 0.722, − 0.507 and − 0.403) in correlation with PLI soil, PLI root and BCF root whereas positive value was only presented by PLI (0.886) given as below in (Table 9). EF root showed positive values (0.516, 0.907 and 1.000) correlation with parameters PLI soil, PLI root and BCF root, respectively mentioned in (Table 9). EF shoot had displayed negative values (− 0.722, − 0.507, − 0.403 and − 0.403 of correlation with parameters PLI soil, PLI root, BCF root and EF root, while on other hand; EF shoot showed positive values (0.886 and 1.000) correlation with PLI shoot and BCF shoot. DIM root showed positive values (0.750, 1.000, 0.907 and 0.907) correlation with parameters PLI soil, PLI shoot, BCF root and EF root whereas showed negative values (− 0.295, − 0.237, − 0.237 and − 0.295) among parameters correlation with PLI shoot, BCF shoot and EF shoot. DIM shoot presented negative values (− 0.383, − 0.295, − 0.237 and − 0.295) of correlation with parameters PLI soil, PLI root, BCF root, EF root and DIM root while positive values (1.000, 0.886, 0.886) showed PLI shoot, BCF shoot and EF shoot. HRI root given positive values (0.750, 1.000, 0.907, 0.907 and 1.000) correlation among parameters PLI soil, PLI root, BCF root, EF root and HRI root while negative values of correlation as (− 0.295, − 0.507, − 0.507, − 0.295) among parameters PLI shoot, BCF shoot, EF shoot and DIM shoot. HRI shoot presented negative correlation value (− 0.383, − 0.295, − 0.237, − 0.237, − 0.295 and − 0.295) among parameters PLI soil, PLI root, BCF root, EF root, DIM root and HRI root whereas positive values (1.000, 0.886, 0.886 and 1.000) correlation among parameters PLI shoot, BCF shoot, EF shoot and DIM shoot, respectively, as given in (Table 9).

**Table 9:** Correlation Cr: PLI (soil), PLI (root), PLI (shoot), BCF (root), BCF (shoot), EF (root), EF (shoot), DIM (root), DIM (shoot), HRI (root), HRI (shoot).

	PLI (Soil)	PLI (Root)	PLI (Shoot)	BCF (Root)	BCF (Shoot)	EF (Root)	EF (shoot)	DIM (Root)	DIM (shoot)	HRI (Root)
PLI (Root)	0.750									
PLI (Shoot)	− 0.383	− 0.295								
BCF (Root)	0.516	0.907	− 0.237							
BCF (Shoot)	− 0.722	− 0.507	0.886	− 0.403						
EF (Root)	0.516	0.907	− 0.237	1.000	− 0.403					
EF (shoot)	− 0.722	− 0.507	0.886	− 0.403	1.000	− 0.403				
DIM (Root)	0.750	1.000	− 0.295	0.907	− 0.507	0.907	− 0.507			
DIM (shoot)	− 0.383	− 0.295	1.000	− 0.237	0.886	− 0.237	0.886	− 0.295		
HRI (Root)	0.750	1.000	− 0.295	0.907	− 0.507	0.907	− 0.507	1.000	− 0.295	
HRI (shoot)	− 0.383	− 0.295	1.000	− 0.237	0.886	− 0.237	0.886	− 0.295	1.000	− 0.295

## Discussions

The type of irrigation source and the district, particularly Pasrur in Sialkot, had noticeable effects for soil (*p* < 0.05) on nickel levels in the soil ranged from 17.57 to 16.06 ppm on average. In the the district Pasrur, irrigation demonstrated significant impacts (*p* < 0.05). The method of heavy metal transfer to different parts of plants is directly linked to the accumulation of these metals in those parts. This transfer method also depends on the plant's characteristics and the amount of heavy metal present in the soil, which might explain the highest concentration of nickel observed in shoot samples^22,23^. The study evaluated nickel contamination in soil using the Pollution Load Index (PLI), which ranged from 0.01 to 0.072. We found higher pollution levels of nickel in Barseem plants, while Barseem plants in Sialkot irrigated with wastewater showed lower pollution levels. Other researchers suggest that the benefits of nickel contamination outweigh the costs^24^, contrary to our findings. Bibi et al.^24^ they reported stronger PLI values for nickel (0.34 and 0.40) at two separate sites compared to our ongoing investigation. This implies that the impact of nickel contamination more significant than what we observed in ours.

A significant relationship (*p* < 0.05) was noticed between the irrigation source of district Sialkot,Tehsil Pasrur was noticed particularly regarding nickel variance in roots. The nickel levels in roots ranged from 11.22 to 4.83. Barseem plant samples from site 3 showed the highest concentration (10.98 ± 0.21), while samples from site 5 had the lowest value (8.93 ± 0.06) among the collected fresh samples. Within the Pasrur irrigation source district, The Bio Concentration Factor (BCF) for nickel ranged from 0.34 to 1.93. Interestingly, Faisalabad corn exhibited the highest transfer rate of nickel, whereas Lahore maize irrigated with wastewater had the lowest percentage. Our findings contrasted with previous results, as discovered that the bio-concentration factor of nickel was higher for all sample types irrigated with various water sources^25^. People can be affected by pollutants in different ways, and one significant concern is heavy metal exposure through the food chain^24^. Recent research shows that all samples analyzed had a Hazardous Risk Index (HRI) for nickel (Ni) values below 1. This means that the presence of these heavy metals didn't pose a risk of nickel toxicity to individuals. The risk index for nickel ranged from 0.011 to 0.091 mg/kg, with Barseem plants showing the highest risk when irrigated with wastewater and the lowest risk when irrigated with canal water. Comparing these findings with previous research by Butt et al.^25^ who reported an HRI for nickel of 0.31, our results show lower risk levels. Interestingly, the HRI for nickel in samples grown with wastewater was even lower at 0.003. This suggests that the method of irrigation, especially using canal water instead of wastewater, may reduce the risk of nickel exposure and toxicity. The Nickel Enrichment Factor (EF) value ranges from 0.04 to 0.26 mg/kg across the samples. The highest and lowest EF values were observed in Barseem plants collected from Sialkot, both of which were irrigated with canal water. This finding indicates that the highest EF for Ni, at 15.26 that was found in Barseem plants from Sialkot region having the irrigated with canal water. The variation in EF values across different samples could be attributed to several factors. One possible reason is the differing sizes of the datasets for all metals in the soil^4^. The exclusion rate of metals and metalloids from the soil may also contribute to these differences. Further research and analysis are needed to better understand the factors influencing the EF values of metals and metalloids in soil samples^4,25^.

The assessment of the health risks through the food chain was a major source of concern in many countries where mining activities were taking place. Intake of heavy metals into the bodies of animals (milk) and humans can occur through a number of different paths, including soil, plants, water, air, and food^26^. However, the food chain was the primary method by which heavy metals enter the bodies of animals and humans^27^. In order to figure out the health risk associated with each heavy metal, it is crucial to assess the quantity of intake by humans and other animals by figuring out the manners in which they came into contact with model organisms. There were a range of ways that exposure had spread to both people and animals, but the food chain was by far the most crucial. Since excessive intake of heavy metals in the diet was associated to a range of developmental defects in both the bodies of animals and people, regular monitoring of these substances in both animals' and humans' diets was essential^26,27^. The irrigation source, Tehsil Pasrur, district Sialkot, does indeed have a significant effect on the dispersion of chromium in water (*p* < 0.05). Chromium concentrations in the water fluctuated throughout different forages in different districts from 40.07 ± 0.49 to 5.56 ± 0.27 ppm. The Pasrur millet that was irrigated with canal water had the lowest concentration of chromium, meanwhile the barseem that also was watered with untreated wastewater had the highest concentration. The main sources of large concentrations of chromium in waste water are from the leather, gilding, tanning, and textile industries. However, leaching from rocks and earth is the natural source of chromium access to ground water aquifers^28,29^.

In Tehsil Pasrur, Sialkot, the ANOVA in shoot places considerable emphasis (*p* < 0.05) on the irrigation source, district, and source under irrigation. The shoot's mean concentration fluctuated from 6.20 ± 0.27 ppm to 2.46 ± 1.00 ppm. Chromium content reported found to be high in millet developed in Sialkot as well as irrigated with sewage water while being low in barseem grown in Pasrur. Compared to the information published by Asdeo^28^ that the concentration of soil in the given forages' seedlings was lower in the ongoing investigation. In contrary to our findings, the outcomes suggested by Khan et al.^14^ were improved. Higher Cr concentrations in the shoot may be caused by heavy metals that demonstrate synergistic effects combining two or more metals, such as Cr, Pb, and Ni^30,31^. The outcomes of the grain ANOVA showed a significant impact (*p* < 0.05) in the district irrigation source in Pasrur. Chromium levels in soil range (P 0.05) from 8.89 ± 0.56 to 1.80 ± 0.34 parts per billion. Low content was found at Barseem site 7 that received sewage water irrigation whereas high content was found in Barseem that received waste water irrigation. In contrast to our findings, Khan et al.^14^ suggested a higher lead concentration. There are discrepancies in the accumulation and translocation capabilities for the same heavy metal, which could explain why it appears that individual plants have distinct functions for accumulating heavy metals^7^, Chromium in soil according to the ongoing investigation is much less than that indicated by Nath et al.^1^.

The district irrigation source Sialkot (Pasrur) demonstrated a significant impact (*p* < 0.05) in the milk ANOVA outcomes. Milk also contains chromium in relatively large quantities from 26.94 ± 1.48 to 0.20 ± 0.09. Barseem plant site 5 (2.10 ± 0.54), which again was irrigated with waste water, had to have a high content, although site 1 (40.07 ± 0.49) had to have a colloidal suspension. Chromium's BCF fluctuated from 1.62 to 15.52. The Barseem site 2 in Pasrur with canal groundwater resources had the highest Cr measurements (40.07 ± 0.49 ppm), while the Barseem in Pasrur with waste water irrigation had the lowest findings. In contrast to the results (0.003 mg/kg) of Zhang et al.^6^ the current study's results significantly higher. Its bio- concentration factor value for Cr in the previous study was greater than just what Verma et al.^31^ had reported. The irrigation source, Tehsil Pasrur, district Sialkot, does indeed have a significant effect on the dispersion of chromium in water (*p* < 0.05). Chromium concentrations in the water fluctuated throughout different forages in different districts from 40.07 ± 0.49 to 5.56 ± 0.27 ppm. The Pasrur millet that was irrigated with canal water had the lowest concentration of chromium, meanwhile the barseem that also was watered with untreated wastewater had the highest concentration. The main sources of large concentrations of chromium in waste water are from the leather, gilding, tanning, and textile industries^15,32^. However, leaching from rocks and earth is the natural source of chromium access to ground water aquifers^1^.

The most prominent factors of soil contamination with heavy metals were waste water irrigation, specifically the use of industrialized water for irrigation, inefficient waste disposal, and excessive pesticide use^32,33^. The predominant fodder crop harvested in Pakistan during the winter months of Rabi, which last from October to March, is Berseem. It is mostly used as a green forage crop and as pallet hay during the off-season. Quantifying human risk along the food chain is significant in countries like Saudi Arabia where wastewater irrigation is still performed without regulation. One of the main aspects that contaminants are subjected to people are through the food chain, which is dependent on contaminated air, water, soil, and food supplies as well as the population that utilizes those things^3,33^. That each heavy metal in our evaluation, except perhaps Cr and Ni, has an HRI value more than 1, which denotes a possible risk to human health from consumption of food, crops in the future. Pb, Cr, and Mn had the maximum HRI values (> 1) in S. oleracea that was watered with effluent, although Cd showed an HRI > 1 in all of the wastewater-irrigated food crops with the exception of *T. aestivum*. Thus according THQ observations, the local population's health may be impacted negatively by Cr which was before in ingested plants^3,18^. Since only a portion of the swallowed heavy metals may be expelled and the remaining money may concentrate in body tissues where they can impair human health, they have postulated that the swallowed dose of heavy metals is not the same as the absorbed pollutant dose. The proposed research is crucial from the standpoint of individual health since it underscores the threat that consuming foods tainted with heavy metals poses to the general community^19^. Found Greater PLI values (3.09–2.56) in soil at two different locations, which is in contradiction to our findings. In soil irrigated with diverse sources, Khan et al.^33^ reported a lower PLI for Ni (2.22–2.10). PLI was found in each sample employed in the current investigation. PLI values were stronger (1.9392–1.8962) at two different locations in the root. Ni levels in roots that were irrigated with diverse sources had a lower PLI (0.1365–1.332), according to reports. Higher PLI values (1.6622–1.7571) were shot at two specific locations. The average daily intake of Ni from human milk approximated 0.0027 to 0.016 mg/kg. Similar daily Ni consumption (0.001–0.009 mg/kg) was found by Khan et al.^34^ in relation to the current experiment. In comparison to this observation, Khan et al.^33^ observed a greater daily Ni intake (0.05 mg/kg). Calculated EDI values were determined to be within permissible limits.

The bio concentration factor was determined by the ratio of the heavy metal concentration in soil to the heavy metal concentration in plant tissue. It was discovered that a wide range of factors, along with the form and concentration of heavy metals in the soil, the soil's physical and chemical properties, a plant's capacity to absorb heavy metals from the soil, and other components, all significantly affected how well plants can absorb heavy metals from the soil. It was considered that heavy metals with BCF values higher than 1 were toxic for both plants and mammals^35^. The PLI value greater than 1 indicated that particular metals had pose a natural event and that specific places required careful inspection in order to manage metal development in the soil^18,19^. These zones did not require respect for these elements, as evidenced by the contamination factor from Cr and Ni being less than 1^35,36^.

## Conclusion

Heavy metal pollution is a serious concern due to its harmful effects on the environment and human health. Implementing stringent monitoring measures and effective pollution control strategies is essential to mitigate the adverse impacts and ensure a sustainable and healthy ecosystem. Nickel (Ni) and chromium (Cr) levels varied significantly in soil, Barseem plant roots and shoots, as well as buffalo milk samples collected from various sites. Site 3 emerged with the highest Ni content in soil and milk samples, while site 4 displayed the highest Cr concentration in both soil and plant shoots. Conversely, site 5 consistently exhibited the lowest levels of Ni and Cr among the sampled sites. The analysis of pollution indices such as PLI, BCF, EF, DIM, HRI, and their correlations provided valuable insights into the accumulation and transfer patterns of heavy metals, shedding light on the complex dynamics of pollution pathways in agricultural ecosystems. These findings emphasize the importance of continuous monitoring and the implementation of effective mitigation strategies to address heavy metal pollution and ensure the protection of environmental and human health in the study area.

## Data Availability

The datasets used and/or analysed during the current study available from the corresponding author on reasonable request.
